# KHSRP combines transcriptional and posttranscriptional mechanisms to regulate monocytic differentiation

**DOI:** 10.1097/BS9.0000000000000122

**Published:** 2022-07-01

**Authors:** Jiayue Xu, Dongsheng Wang, Hongliu Ma, Xueying Zhai, Yue Huo, Yue Ren, Weiqian Li, Le Chang, Dongxu Lu, Yuehong Guo, Yanmin Si, Yufeng Gao, Xiaoshuang Wang, Yanni Ma, Fang Wang, Jia Yu

**Affiliations:** a State Key Laboratory of Medical Molecular Biology, Haihe Laboratory of Cell Ecosystem, Institute of Basic Medical Sciences, Chinese Academy of Medical Sciences, School of Basic Medicine Peking Union Medical College, Beijing 100005, China; b Key Laboratory of RNA and Hematopoietic Regulation, Chinese Academy of Medical Sciences, Beijing 100005, China; c Department of Clinical Laboratory, Sichuan Cancer Hospital and Institute, Sichuan Cancer Center, School of Medicine, University of Electronic Science and Technology of China, Chengdu 610041, China; d Department of Gynecology, Dian Jiang People’s Hospital of Chong Qing, Chongqing 408300, China; e Medical Epigenetic Research Center, Chinese Academy of Medical Sciences, Beijing 100005, China

**Keywords:** KHSRP, Monocytic differentiation, RNA-binding proteins, Transcriptional regulation

## Abstract

RNA-binding proteins (RBPs) are widely involved in the transcriptional and posttranscriptional regulation of multiple biological processes. The transcriptional regulatory ability of RBPs was indicated by the identification of chromatin-enriched RBPs (Che-RBPs). One of these proteins, KH-type splicing regulatory protein (KHSRP), is a multifunctional RBP that has been implicated in mRNA decay, alternative splicing, and miRNA biogenesis and plays an essential role in myeloid differentiation by facilitating the maturation of miR-129. In this study, we revealed that KHSRP regulates monocytic differentiation by regulating gene transcription and RNA splicing. KHSRP-occupied specific genomic sites in promoter and enhancer regions to regulate the expression of several hematopoietic genes through transcriptional activation and bound to pre-mRNA intronic regions to modulate alternative splicing during monocytic differentiation. Of note, KHSRP had co-regulatory effects at both the transcriptional and posttranscriptional levels on MOGOH and ADARB1. Taken together, our analyses revealed the dual DNA- and RNA-binding activities of KHSRP and have provided a paradigm to guide the analysis of other functional Che-RBPs in different biological systems.

## 1. INTRODUCTION

RNA-binding proteins (RBPs) are generally composed of small RNA-binding domains (RBDs) such as the RNA-recognition motif,^1^ double-stranded RBD,^2^ zinc-finger domains,^3^ and the human heterogeneous nuclear ribonucleoprotein K homology, or KH, domain.^4^ This important class of proteins is widely involved in transcriptional and posttranscriptional gene regulation by binding specifically to well-defined RBDs and through RBP-RNA interactions^1,2^ to mediate processes such as alternative splicing (AS), transport, modification, editing, decay, and translation.^5,6^ In fact, many RBPs participate in more than one of these processes. For example, the mammalian RBP Nova modulates both AS and poly(A) site usage.^3^ Moreover, recent studies have revealed that RBPs not only play important roles in posttranscriptional regulation but also interact with chromatin to regulate gene transcription. The chromatin-enriched RBP (Che-RBP) QKI5 regulates RNA processing and functions as a novel transcriptional activator during monocytic differentiation.^7^ RBP Lin28A binds to genes in the proximity of transcription start sites and recruits Tet methylcytosine dioxygenase 1 (Tet1) to control gene transcription in mouse embryonic stem cells.^8^ RNA methyltransferase-like 3 was shown to be recruited to chromatin by the transcription factor CRPBPZ to induce m6A modification of associated mRNAs in a human leukemia cell line (MOLM13).^9^ The WD repeat domain 43 (WDR43) is recruited to promoters by noncoding/nascent RNAs to release Pol II, thereby facilitating transcriptional elongation in embryonic stem cells.^10^ In addition, chromatin immunoprecipitation sequencing (ChIP-seq) analysis of K562 and HepG2 cells showed that multiple nuclear RBPs interact with chromatin to regulate gene transcription.^11,12^ These regulatory roles are essential to both physiological and disease states, with defects in RBP function causing diverse genetic alterations and abnormal cell differentiation, leading to conditions such as neurodegeneration, autoimmunity, and cancer.^4,13–18^

Using mass-spectrometry-based methods, hundreds of proteins that bind to RNA in human and mouse cells have been identified,^1,19–21^ and their genes account for between 6% and 8% of all protein-encoding genes. Although many types of RBPs have been identified, the functions of only a few have been fully elucidated, and a dissection of RBP-RNA regulatory networks through the integration of multiple data types is essential. Such in-depth research into RBPs has been made possible by the continuous progress of technologies such as in vivo binding assays. For example, crosslinking immunoprecipitation sequencing (CLIP-seq) provides a set of candidate functional elements directly bound to each RBP, and integration of the results with knockdown/RNA-seq profiles can indicate RNA expression and regulatory splicing patterns. ChIP-seq profiles of DNA associations offer researchers the opportunity to understand the complex interconnectivity between chromatin association and RNA processing. Such integrated analyses may facilitate the identification of the roles of RBPs in broader cellular regulatory networks. Ren et al revealed that approximately 9.6% (52/544) of all annotated RBPs are commonly chromatin-enriched, and the chromatin-binding capacity of these proteins may represent a previously under-appreciated layer of gene expression regulation.^7^ They also defined 7 hematopoiesis-related Che-RBPs (ADAR, PTBP3, KHSRP, ELAVL1, NUDT21, SETD1A, and QKI5) and illustrated a novel chromatin-associating function for QKI5, which is located at the genomic loci of several target genes in monocytic cells and activates their transcription. Such genes include *CXCL2*, which encodes a cytokine that is essential for monocytic differentiation.^7,22–24^

In this study, we concentrated on a hematopoiesis-related Che-RBP, KH-type splicing regulatory protein (KHSRP), which is a multifunctional, single-stranded nucleic acid (DNA or RNA)-binding protein.^25^ KHSRP is important because it regulates both transcriptional and posttranscriptional processes. KHSRP was reported to bind to a sequence far upstream of the myc promoter to control^26^ and regulate TNF-α promoter activity.^27^ Additionally, several recent studies revealed that KHSRP is an essential factor for AU-rich element-directed mRNA decay^28^ and is a novel regulatory protein that mediates exon inclusion via an intronic splicing enhancer.^25^ RBPs that regulate translation are also known to contribute to the development and function of hematological lineages, as well as hematological malignancies, by acting as nodes in multiple signaling pathways.^29^ However, the mechanisms by which RBPs such as KHSRP regulate hematopoietic processes are still poorly understood. Previous studies revealed that granulocyte differentiation is promoted by the KHSRP^−^miR-129-RUNX1 regulatory axis at the expense of monocyte-macrophage differentiation.^25^ Consequently, a comprehensive understanding of the chromatin-binding capacity and chromatin-associated functions of KHSRP, as well as the mechanism by which it regulates mononuclear differentiation, may provide a new perspective on the role of Che-RBPs in monocytic differentiation.

As a classical Che-RBP, we found that KHSRP not only performed a splicing regulatory function in posttranscriptional processes but also activated the transcription of several genes associated with monocytic differentiation. Therefore, our study indicated that, during monocytic differentiation, KHSRP performs regulatory functions that are mediated at both the transcriptional and posttranscriptional levels. These findings provide a paradigm that can be used to guide the analysis of other functional Che-RBPs in different biological systems.

## 2. RESULTS

### 2.1. KHSRP is associated with RNA and regulates alternative RNA splicing

**Figure 1.**
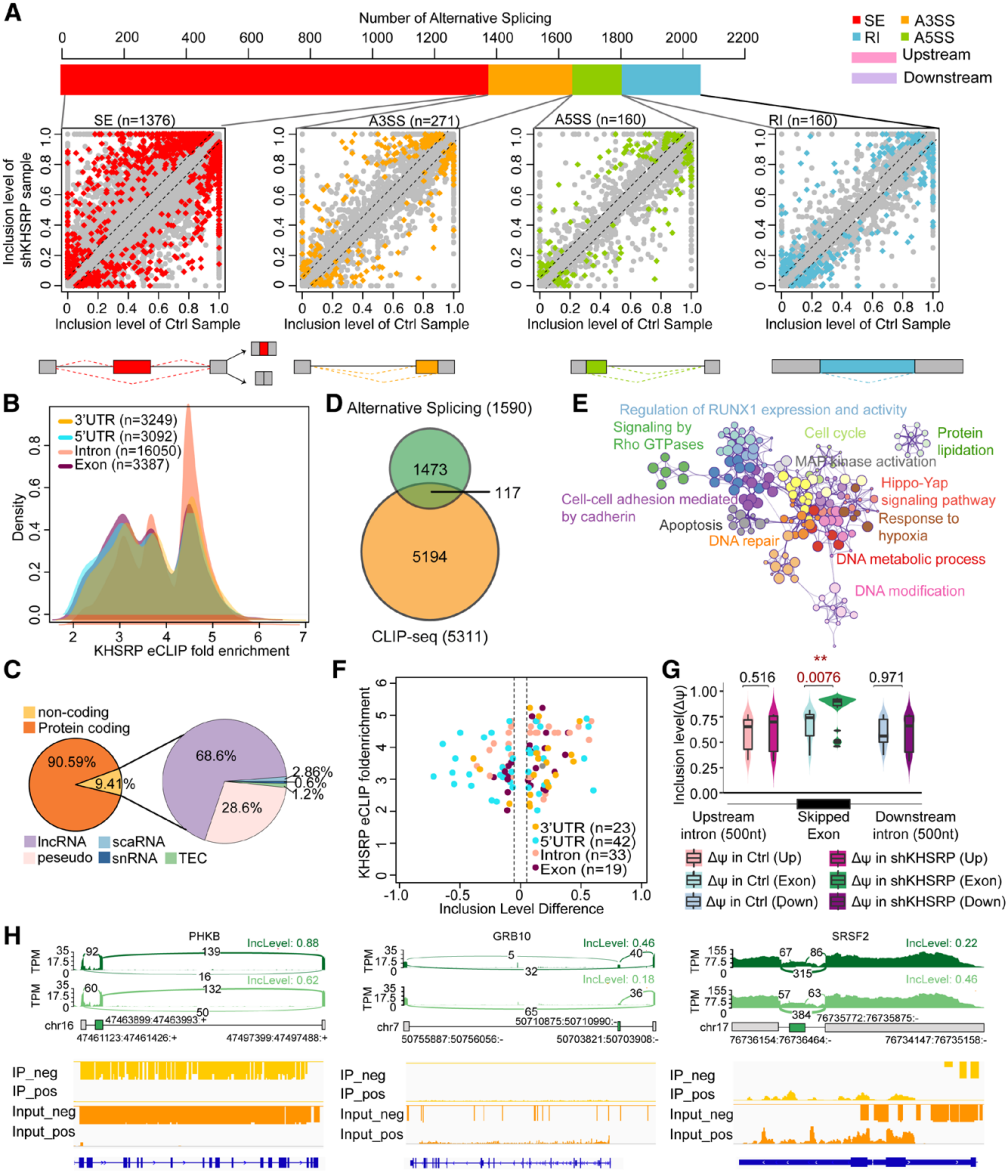
Identification of splicing regulatory patterns by integration of CLIP-seq and RNA-seq datasets (A) Plot indicating the number of AS events (upper panel). Identification of 4 major AS patterns by comparison of shKHSRP and Ctrl RNA-seq data; skipped exon (SE, red), alternative 3’ splice site (A3SS, orange), alternative 5’ splice site (A5SS, green), and retained intron (RI, blue). (B) Density of KHSRP eCLIP region-level enrichment in 3’-UTR, CDS, intron, and exon regions. (C) Pie chart showing the proportion of protein-coding genes (orange) and nonprotein coding genes (yellow), including lncRNA (purple), pseudo gene (pink), scaRNA (light blue), snRNA (dark blue), and TEC (light green), that were associated with KHSRP. (D) Venn diagram showing the region of intersection of AS events, including those that were absent or present following *KHSRP* knockdown and the eCLIP enriched region. (E) Enrichment network representing the top-12 enriched terms for AS events located in eCLIP enriched region. (F) Plot showing AS events following *KHSRP* knockdown (x-axis) vs (y-axis) fold enrichment in KHSRP eCLIP for the indicated transcript-binding regions. (G) Distribution of ΔΨ changes following *KHSRP* knockdown in the eCLIP enrichment region (*P* < .05 indicates statistical significance by Wilcoxon rank-sum test). (H) Sashimi plots showing differential AS events in shKHSRP (dark green) and control (light green) samples (upper panel). Alternatively, spliced exons are shown in green. Bars indicate eCLIP signals on gene loci in the IP (yellow) and input (orange) (lower panel).

To clarify the regulatory effects of KHSRP on RNA splicing, we first identified the overall changes in the splicing profile caused by KHSRP knockdown (Fig. 1A and Supplementary Table 1, https://doi.org/10.5281/zenodo.6496559). In total, we identified 2061 AS events classified into 4 patterns as follows: 1376 skipped exons (SEs), mutually exclusive exons (MXEs), 271 alternative 3’ splice sites (A3SSs), 160 alternative 5’ splice sites (A5SSs), and 254 retained introns (RIs) (Supplementary Table 2, https://doi.org/10.5281/zenodo.6496559). The eCLIP results showed that KHSRP tended to be associated with introns (16,050, 62.26%), with an average 3.98-fold enrichment, suggesting it binds to the intronic splicing elements to modulate AS (Fig. 1B). Subsequently, by comparing the changes in eCLIP and AS events following KHSRP knockdown, we obtained 112 targets with splicing patterns that might be controlled by KHSRP at the posttranscriptional level (Fig. 1C and Supplementary Table 3, https://doi.org/10.5281/zenodo.6496559). The gene annotation results showed that their functions were mainly enriched in terms related to “cell cycle,” “DNA repair,” “response to hypoxia,” and “cell-cell adhesion mediated by cadherin” (Fig. 1D). Additionally, the inclusion of AS events tended to increase following KHSRP knockdown, suggesting that alternative exon inclusion is suppressed preferentially (Fig. 1E). Moreover, directly bound KHSRP ASs conferred stronger regulation than other types (Fig. 1F), such as KHSRP direct splicing targets, including phosphorylase kinase regulatory subunit beta, growth factor receptor bound protein 10, and serine and arginine rich splicing factor 2 (Fig. 1G).

### 2.2. Che-RBP KHSRP regulates gene transcription

**Figure 2.**
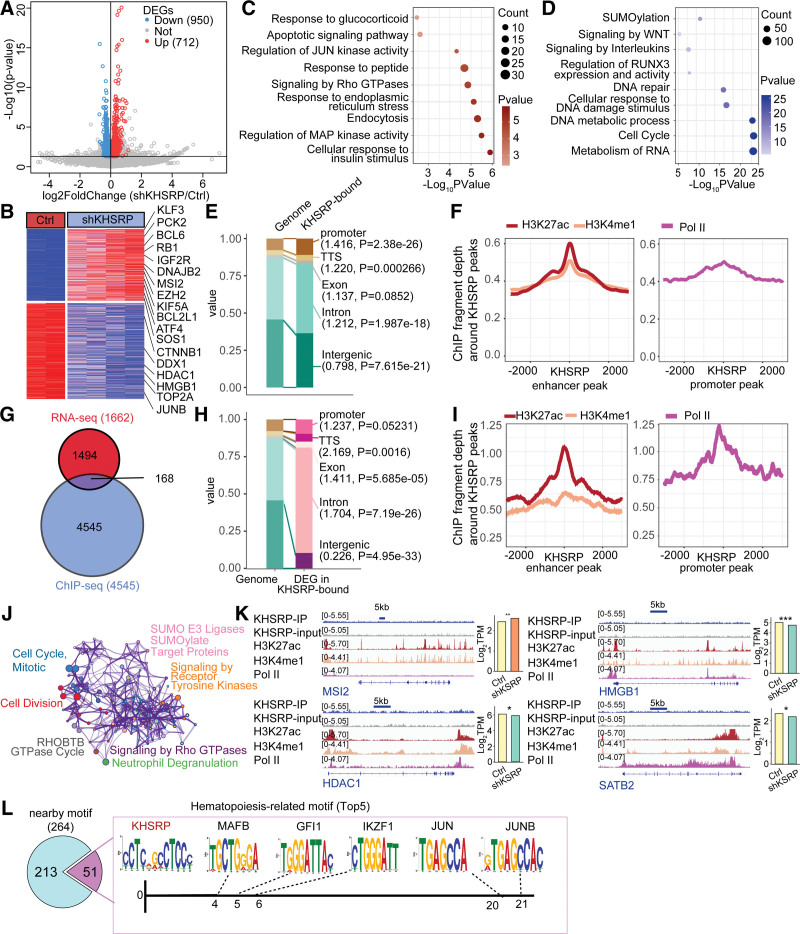
Integration of ChIP-seq and RNA-seq data identifies KHSRPs that interact with chromatin and activate gene transcription. (A, B) Volcano plot (A) and heatmap (B) showing differentially expressed genes between shKHSRP and control samples. (C, D) GO functional enrichment analysis of activated (C) and repressed genes (D) in shKHSRP sample compared with control sample. (E) Percentages of different genic and intergenic regions associated with KHSRP peaks identified by ChIP-seq in THP-1 cells (KHSRP-bound). The abundance of each type of region in the human genome (Genome) is shown for comparison. The numbers in parentheses indicate the enrichment ratio relative to the genome; *P* values were determined by single-tailed Fisher’s exact test (*P < .05). (F) Metaplot showing the distribution of H3K4me1 and H3K27ac ChIP-seq (left panel) and Pol II ChIP-seq fragment depth (right panel) within the −3000 bp to +3000 bp region around the KHSRP ChIP-seq peaks in the enhancer and promoter regions of 168 transcriptionally activated genes. (G) Venn diagram illustrating the intersection of KHSRP-bound genes and KHSRP-activated genes. (H) Percentages of different genic and intergenic regions associated with KHSRP peaks as identified by ChIP-seq in 168 KHSRP transcriptionally activated genes. (I) Metaplot showing the distribution of H3K4me1 and H3K27ac ChIP-seq (left panel) and Pol II ChIP-seq fragment depths (right panel) within the region −3000 bp to +3000 bp around the KHSRP ChIP-seq peaks in the enhancer and promoter regions of 168 transcriptionally activated genes. (J) Enrichment network representing the top 10 enriched terms of 168 KHSRP transcriptionally activated genes. (K) Genomic visualization of KHSRP, H3K4me1, K3H27ac, and Pol II ChIP-seq datasets at the indicated gene loci. (L) Nearby TF-binding motif prediction by SpaMo was used to identify putative hematopoiesis-related interaction partner TFs for KHSRP. The top-5 identified TFs determined by e-value are shown in the figure. The number under the x-axis represents the best gap between the KHSRP motif and indicated TF motifs.

To investigate the potential transcriptional regulatory activity of KHSRP, we first analyzed the differentially expressed genes (DEGs) induced by *KHSRP* knockdown. We identified 1662 DEGs, of which 950 were downregulated and 712 were upregulated (Fig. 2A and Supplementary Table 4, https://doi.org/10.5281/zenodo.6496559). The downregulated DEGs included several hematopoiesis-related genes, such as *JUNB*, *HMGB1*, and *HDAC1*, while genes related to transcription factors (*BCL6* and *KLF3*), the cell cycle (*RB1* and *CTNNB1*), and epigenetics (*EZH2*) were significantly upregulated (Fig. 2B and Supplementary Table 4, https://doi.org/10.5281/zenodo.6496559), which supports the repressive roles of KHSRP during monocyte differentiation. Functional enrichment analysis of the shKHSRP vs control groups showed the over-representation of cell-response terms, such as responses to glucocorticoid, peptides, endoplasmic reticulum stress, and cell proliferation terms, including regulation of JUN kinase activity (Fig. 2C and Supplementary Table 4, https://doi.org/10.5281/zenodo.6496559). In contrast, the downregulated DEGs in the shKHSRP group were mainly clustered around DNA repair and cell cycle terms (Fig. 2D and Supplementary Table 4, https://doi.org/10.5281/zenodo.6496559).

### 2.3. KHSRP preferentially binds to activated gene promoters and introns

Recent studies have suggested that the association between RBPs and chromatin may be essential for the regulation of transcription.^30,31^ We previously identified the Che-RBP KHSRP in monocytic cells; therefore, in the current study we aimed to determine how it is associated with chromatin. ChIP-seq analysis showed that KHSRP was mainly localized in genic regions (Fig. 2E) and was over-represented within gene promoters, introns, and transcription termination sites compared with the abundance of these regions in the human genome (Fig. 2E). To assess the potential transcriptional regulatory activity of KHSRP, we undertook a further investigation of the chromatin landscape of KHSRP-bound promoters and enhancers. We found that the active enhancer marker methylated H3K4 (H3K4me1) and the active promoter marker acetylated H3K27 (H3K27ac) aggregated around KHSRP-bound enhancers and promoters, respectively (Fig. 2F, left panel). Additionally, Pol II signals also aggregated around KHSRP-bound promoters (Fig. 2F, right panel). These results indicated that KHSRP preferentially binds to active gene promoters and introns. Furthermore, by comparing the list of DEGs detected following KHSRP knockdown with the KHSRP-bound genes revealed by ChIP-seq, we identified 168 genes potentially transcriptionally regulated by KHSRP (Fig. 2G and Supplementary Table 3, https://doi.org/10.5281/zenodo.6496559). KHSRP tended to be associated with the intronic regions of these 168 targets (*P* = 7.19e-26) (Fig. 2H). Notably, the active promoter markers H3K27ac and Pol II were more enriched in the 168 gene regions, suggesting the stronger transcriptional regulation activity of KHSRP on its direct targets (Fig. 2I). These 168 targets participated in multiple biological processes, including “cell cycle, mitotic,” “cell division,” “signaling by Rho GTPases,” and “neutrophil degranulation” (Fig. 2J). We selected several KHSRP-occupied genes (including Musashi RNA-binding protein 2, histone deacetylase 1, high-mobility group box 1 [*HMGB1*], and SATB homeobox 2) that also colocalized with the H3K4me3 and Pol II signals. Genomic visualization of the KHSRP, H3K4me1, K3H27ac, and Pol II ChIP-seq datasets on the indicated gene loci is shown in Figure 2K. In addition, to determine whether KHSRP is recruited by other chromatin-interacting proteins, such as TFs, we first screened for TF-binding motifs located nearby the KHSRP-binding motif. We identified 264 distinct DNA elements within 150 bp of the KHSRP ChIP motif (CCTCRGCCTCCC). Among them, 51 were annotated as hematopoiesis-related TF-binding sites, including MAF bZIP transcription factor B, growth factor independent 1 transcriptional repressor, AP-1 (*JUN*), DNA-binding protein Ikaros (*IKZF1*), and Jun-B (*JUNB*), suggesting that KHSRP is recruited by these TFs (Fig. 2L and Supplementary Table 3, https://doi.org/10.5281/zenodo.6496559).

### 2.4. Dual DNA- and RNA-binding activities of KHSRP

**Figure 3.**
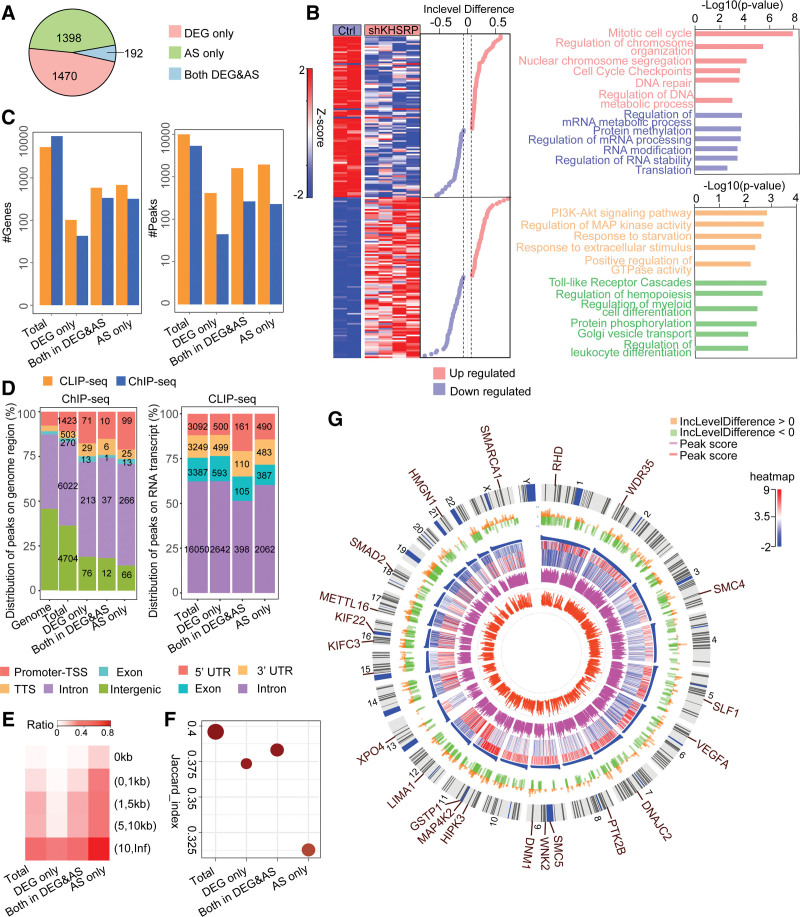
Comparative analysis of transcriptional and posttranscriptional functions (A) Number of differentially expressed genes without AS events between shKHSRP and control samples (DEG-only group), genes with AS events without DEG between shKHSRP and control samples (AS-only group), and differentially expressed genes with AS events between shKHSRP and control samples (Both group). (B) Heatmap displaying expression level of 192 Both group genes (left) and difference inclusion levels (DILs) of relative AS events (middle). Bar plot showing enriched terms in the 4 groups (right) that were downregulated in DEG and upregulated in DIL (pink), downregulated in DEG and downregulated in DIL (blue), upregulated in DEG and upregulated in DIL (orange), or downregulated in DEG and upregulated in DIL (green). (C) Comparison of occupied gene numbers (left) and peak numbers (right) between ChIP-seq and CLIP-seq datasets of the 4 groups: all enrichment genes on the genome (Total), DEG-only group, AS-only group, and Both group. (D) ChIP-seq signal distribution (left) and CLIP-seq signal distribution (right) with human genomic intrinsic constitution in a comparison of the 4 groups (Total, DEG-only, AS-only, and Both). (E) Heatmap presenting the occupation ratio of histone marker ChIP signals colocalized with ChIP peaks at promoter and gene body regions of co-occupied genes in the 4 groups (Total, DEG-only, AS-only, and Both). (F) Comparison of co-occupied genes from ChIP-seq and CLIP-seq datasets of the 4 groups (Total, DEG-only, AS-only, and Both). The x-axis shows the Jaccard index of the ChIP-seq and CLIP-seq occupied genes of each group; bubble size indicates co-occupied gene number. (G) Circos plot describing the overall multi-layered relationship of whole genomes; bars represent the DIL of AS events (DIL > 0, orange; DIL < 0, green), peak scores for chromatin interactions (red), and CLIP-seq signals (pink), and the heatmap displays the expression levels of the shKHSRP and control samples.

To compare the transcriptional and posttranscriptional regulatory activities of the Che-RBP KHSRP, we compared the 1662 DEGs and 2061 changed AS events (1590 genes) identified following KHSRP knockdown and divided them into 3 groups: 1470 DEGs without changes to AS events (DEG-only genes), 1398 genes without differential expression but with changes to AS events (AS-only genes), and 192 DEGs with changes to AS (Both) (Fig. 3A and Supplementary Table 5, https://doi.org/10.5281/zenodo.6496559). The 192 DEGs with changes to AS events were selected for further analysis and divided into 4 subgroups (Fig. 3B and Supplementary Table 5, https://doi.org/10.5281/zenodo.6496559). Subgroup 1 comprised 58 genes that were downregulated and had increased alternative exon inclusion following KHSRP knockdown; GO enrichment analysis showed that these genes were significantly associated with processes such as “cell cycle” and “DNA repair.” Subgroup 2 comprised 38 genes that were downregulated and had reduced alternative exon exclusion following KHSRP knockdown; these genes were shown to participate in several functions, including “regulation of mRNA processing,” “RNA modification,” and “translation.” Subgroup 3 contained 49 upregulated genes that had increased alternative exon inclusion clustered mainly around processes such as “PI3K-Akt signaling pathway” and “regulation of MAP kinase activity.” Subgroup 4 contained 47 upregulated genes that had increased alternative exon exclusion; the genes were enriched in terms such as “regulation of hemopoiesis” and “regulation of leukocyte differentiation.”

Having identified the binding patterns of the Che-RBP KHSRP across DNA and RNA targets, we next explored whether KHSRP displays a preference for genomic or RNA targets by comparing the ChIP-seq and CLIP-seq data of the 3 groups (DEG-only, AS-only, and Both). We found that both the KHSRP peaks and gene numbers displayed similar enrichment tendencies on DNA and RNA, suggesting balanced regulatory features and no significant differences among the different groups (Fig. 3C).^7^ Then, we investigated the KHSRP ChIP-seq and CLIP-seq data to identify the possible regulatory functions of KHSRP. ChIP-seq analysis revealed that all 3 KHSRP groups tended to bind to protein-coding gene regions, and all were over-represented in promoter regions compared with the average promoter abundance of the human genome (Fig. 3D, left panel). In addition, previous studies revealed that KHSRP regulates intronic splicing and mRNA decay in the 3′-UTR, with clear 3′-UTR and intron-binding preference in HepG2 cells and K562 cells.^32,33^ Our KHSRP CLIP-seq results for THP-1 cells were consistent with previous reports showing predominant enrichment in intronic regions (Fig. 3D, right panel). It is worth mentioning that the ChIP-seq data revealed fewer binding sites in the promoter regions of genes in the Both group compared with the numbers detected in the other groups, while the CLIP-seq data indicated that the genes in the Both group exhibited a greater preference for binding to the 5′-UTR than those in the other groups. This may be due to fact that KHSRP transitions from the chromatin to nascent transcripts to accomplish its dual DNA- and RNA-regulating activities. We calculated the relative positions of the peaks in the ChIP-seq and CLIP-seq data for the subgroups to explore the interactions between KHSRP DNA and RNA binding. We found similar trends among the 4 subgroups, in that the overlapping rates between the ChIP and CLIP peaks of each subgroup were very low (only .294%, .457%, 0%, and .452% in the KHSRP total, DEG-only, Both, and AS-only groups, respectively) (Fig. 3E). CLIP peaks only appeared concurrently in the range of 5–10 kb or beyond 10 kb relative to the ChIP peaks (3.86% and 90.55% in the KHSRP total subgroup, 4.11% and 89.28% in the DEG-only subgroup, 6.04% and 86.79% in the Both subgroup, and 3.87% and 89.86% in the AS-only subgroup) (Fig. 3E). These results suggested that KHSRP interacts with chromatin or RNA in unrelated ways. To further clarify details of the co-occupation of genes by KHSRP at both the DNA and RNA levels, we analyzed the overlapping rates of gene-binding by calculating the “Jaccard” index (Fig. 3F). This analysis revealed a relatively high overlap rate between the ChIP- and CLIP-target genes, indicating that KHSRP has multi-layered roles in the regulation of gene expression. The overlap rate of genes in the Both group was higher than those of the other 2 groups (DEG-only and AS-only), suggesting that this subgroup contains a greater proportion of genes involved in co-regulation. Notably, KHSRP-regulated the expression of multiple genes at both the transcriptional and posttranscriptional levels in monocytes, including chromatin-related genes [high-mobility group nucleosome binding domain 1 (*HMGN1*); SWI/SNF-related, matrix-associated, actin-dependent regulator of chromatin, subfamily a, member 1 (*SMARCA1*); and structural maintenance of chromosomes 4 and 5 (*SMC4*, *SMC5*)], protein kinases [protein tyrosine kinase 2 beta (PTK2B), homeodomain interacting protein kinase 3 (*HIPK3*), WNK lysine deficient protein kinase 2 (*WNK2*), and mitogen-activated protein kinase kinase kinase kinase 2 (*MAP4K2*)], hematopoiesis-related genes [vascular endothelial growth factor A (*VEGFA*), DNAJ heat shock protein family (*Hsp40*) member C2 (*DNAJC2*), and Rh blood group D antigen (*RHD*)], epigenetic-related genes [SMC5-SMC6 complex localization factor 1 (*SLF1*) and methyltransferase 16, N6-methyladenosine (*METTL16*)], kinesin family and dynamin members [dynamin 1 (*DNM1*), kinesin family member C3 (*KLFC3*), and kinesin family member 22 (*KIF22*)], and genes associated with cellular processes [WD repeat domain 35 (*WDR35*) and SMAD family member 2 (*SMAD2*), glutathione S-transferase Pi 1 (*GSTP1*), LIM domain and actin binding 1 (*LIMA1*), and exportin 4 (*XPO4*)] (Fig. 3G), suggesting that these important functional genes cooperate in various ways at both the transcriptional and transcriptional level.

### 2.5. KHSRP participates in monocyte differentiation by regulating AS

**Figure 4.**
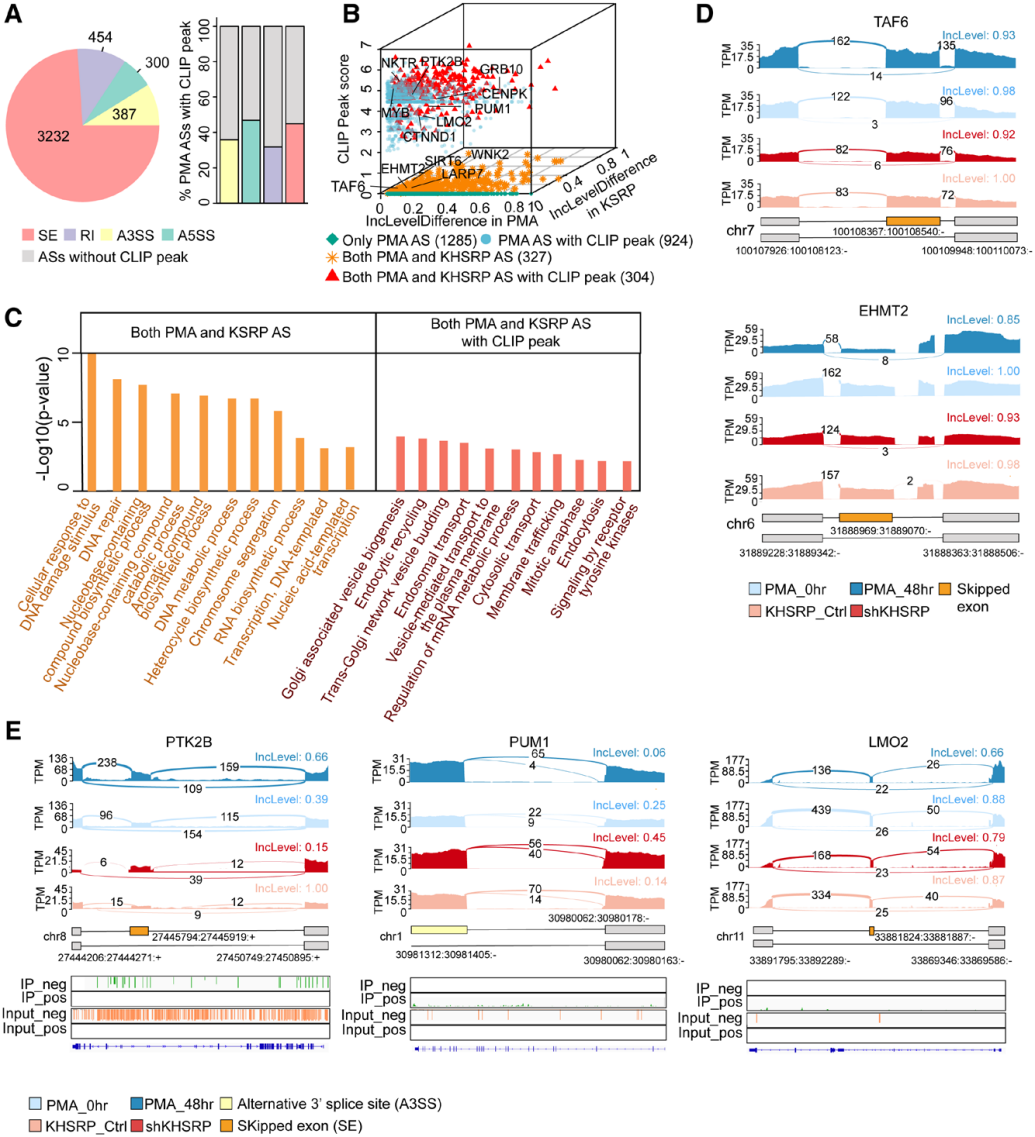
KHSRP regulates alternative splicing during monocyte differentiation (A) Pie chart showing the proportion of major patterns of AS events following PMA treatment (left panel) and proportion of eCLIP enriched regions. (B) 3D scatterplot presenting the relationship among AS events following PMA treatment and following KHSRP and eCLIP enrichment. (C) GO functional enrichment analysis of AS events following PMA treatment and *KHSRP* knockdown and PMA treatment combined with KHSRP knockdown with eCLIP peaks. (D) Sashimi plots showing differential AS events both in the shKHSRP sample (red) and control sample (pink) and in PMA-treated samples (PMA 0 h and 48 h shown in light and dark blue, respectively). Alternatively, spliced exons are shown in orange. (E) Sashimi plots showing differential AS events both in shKHSRP- and PMA-treated samples (upper panel); bars indicate eCLIP signals on gene loci in IP (green) and input (orange) (lower panel).

Recent transcriptomic studies have characterized AS events in hematopoietic stem cells^34^ as well as the processes of erythropoiesis,^35,36^ terminal murine granulopoiesis,^37^ megakaryopoiesis,^35^ and monocyte-to-macrophage differentiation.^38^ To define the posttranscriptional regulation of KHSRP in monocytic differentiation, we detected differential AS events during the monopoiesis of PMA-treated THP-1 cells (PMA-0 h and PMA-48h) and found 4373 DASEs in 2839 genes (Fig. 4A left panel, Supplementary Table 1, https://doi.org/10.5281/zenodo.6496559). The most common types of DASEs in monocytic differentiation were cassette exons (3232) and intron retention (454) and alternative 5′ (300) or 3′ splice sites (387), which were detected at similar levels (Fig. 4A left panel, Supplementary Table 6, https://doi.org/10.5281/zenodo.6496559). Among them, we found that 44.86% of SEs, 31.76% of RIs, 47.23% of A5SS, and 35.85% of A3SS were associated with KHSRP eCLIP peaks, accounting for nearly 40% (924) of DASEs (Fig. 4A right panel, 4B, Supplementary Table 6, https://doi.org/10.5281/zenodo.6496559). We integrated the relationships among AS events in PMA-treated samples, AS events following KHSRP knockdown, and eCLIP enrichment (Fig. 4B, Supplementary Table 7, https://doi.org/10.5281/zenodo.6496559). The 327 DASEs that were detected following both shKHSRP knockdown and PMA treatment were enriched in processes such as “cellular response to DNA damage stimulus”, “DNA repair”, “RNA biosynthetic process”, and “chromosome segregation” (Fig. 4B, C, Supplementary Table 7, https://doi.org/10.5281/zenodo.6496559). These genes included TATA-box-binding protein-associated factor 6 (*TAF6*), a binding protein that may participate in basal transcription and serve as a co-activator in promoter recognition and transcription initiation, and euchromatic histone lysine methyltransferase 2 (*EHMT2*), which encodes the enzyme responsible for catalyzing histone H3 methylation at lysine 9, leading to the recruitment of additional epigenetic regulators and transcription repression (Fig. 4D).^39,40^ TAF6 plays important regulatory roles in human monocytes and macrophages,^41^ suggesting that KHSRP might regulate monopoiesis by regulating the splicing of TAF6. Moreover, 304 of these overlapping DASEs were also enriched in the CLIP-seq data and were mainly gathered around “Golgi-associated vesicle biogenesis”, “endocytic recycling”, “trans-Golgi network vesicle budding”, and “endosomal transport” (Fig. 4B and C). For example, A protein kinase, protein tyrosine kinase, *PTK2B*, Pumilio RNA binding family member 1 (*PUM1*), and LIM domain only 2 (*LMO2*) (Fig. 4E) all exhibited differential AS events during monocyte differentiation.^41^

### 2.6. KHSRP participates in monocyte differentiation by regulating gene transcription

**Figure 5.**
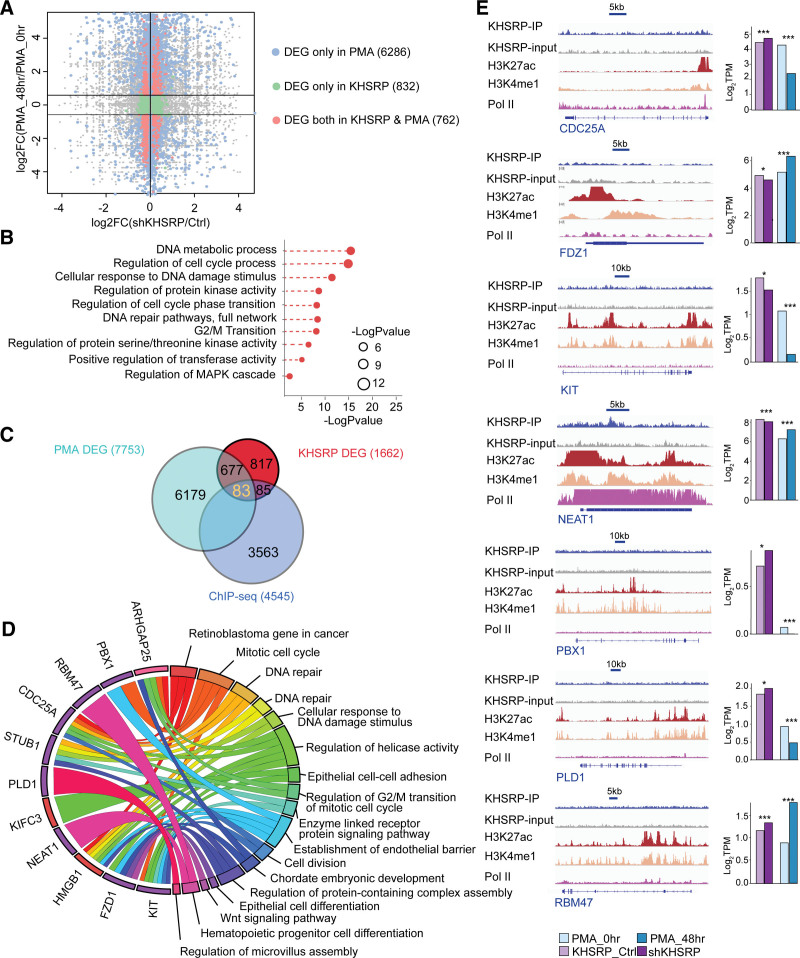
KHSRP participates in the regulation of monocyte differentiation at the transcriptional level (A) Scatterplot showing comparison of DEGs between the shKHSRP and control samples and the PMA-treated and control samples. (B) GO functional enrichment analysis of DEGs commonly affected by KHSRP and PMA. (C) Venn diagram showing common DEGs between the shKHSRP and control samples, DEGs between the PMA-treated and control samples, and genes with KHSRP peaks identified by ChIP-seq. (D) Circos plot showing genes and GO enrichment of DEGs affected by both shKHSRP and PMA, with enrichment peaks identified by ChIP-seq. (E) Genomic visualization of KHSRP, H3K27ac, H3K4me1, and Pol II ChIP-seq datasets for the indicated gene loci (left panel), and bar plot showing expression levels in the shKHSRP group compared with the control group and the PMA group compared with the control group.

It has been reported that the attenuation of KHSRP expression is required for monocyte differentiation.^42^ To define the influence of KHSRP on monocytic differentiation at the transcriptional level, we compared the DEGs caused by KHSRP knockdown with those detected following PMA treatment (Fig. 5A and Supplementary Table 8, https://doi.org/10.5281/zenodo.6496559) and found that 762 overlapped and accounted for nearly half of the KHSRP-regulated DEGs. Among them, 513 genes (317 commonly repressed and 196 commonly activated) were commonly affected by KHSRP knockdown and PMA treatment or were enriched in several monopoiesis-related pathways, such as “regulation of cell cycle phase transition,” “G2/M transition,” and “regulation of protein kinase activity,” supporting a regulatory function of KHSRP during monocytic differentiation (Fig. 5B). Additionally, by comparing the common DEGs with the KHSRP-occupied genes revealed by ChIP-seq, we found that 83 genes (16.11% of the common DEGs) were transcriptionally regulated by KHSRP (Fig. 5C). Functional enrichment analysis of these genes showed the over-representation of hematopoiesis-relevant terms, including “hematopoietic progenitor cell differentiation,” “Wnt signaling pathway,” and “epithelial cell differentiation” (Fig. 5D). These genes are implicated in some essential processes in monocytic differentiation (Fig. 5E), such as hematopoiesis-related genes (*CDC25A, HMGB1, KIT, NEAT1*, PBX homeobox1 [*PBX1*]), phospholipase D1 [*PLD1*]) and RBPs (RNA-binding motif protein 47). Taken together, these results indicate that KHSRP modulates hematopoietic gene expression at the transcriptional level during monocytic differentiation.

### 2.7. KHSRP combines transcriptional and posttranscriptional regulation during monocyte differentiation

**Figure 6.**
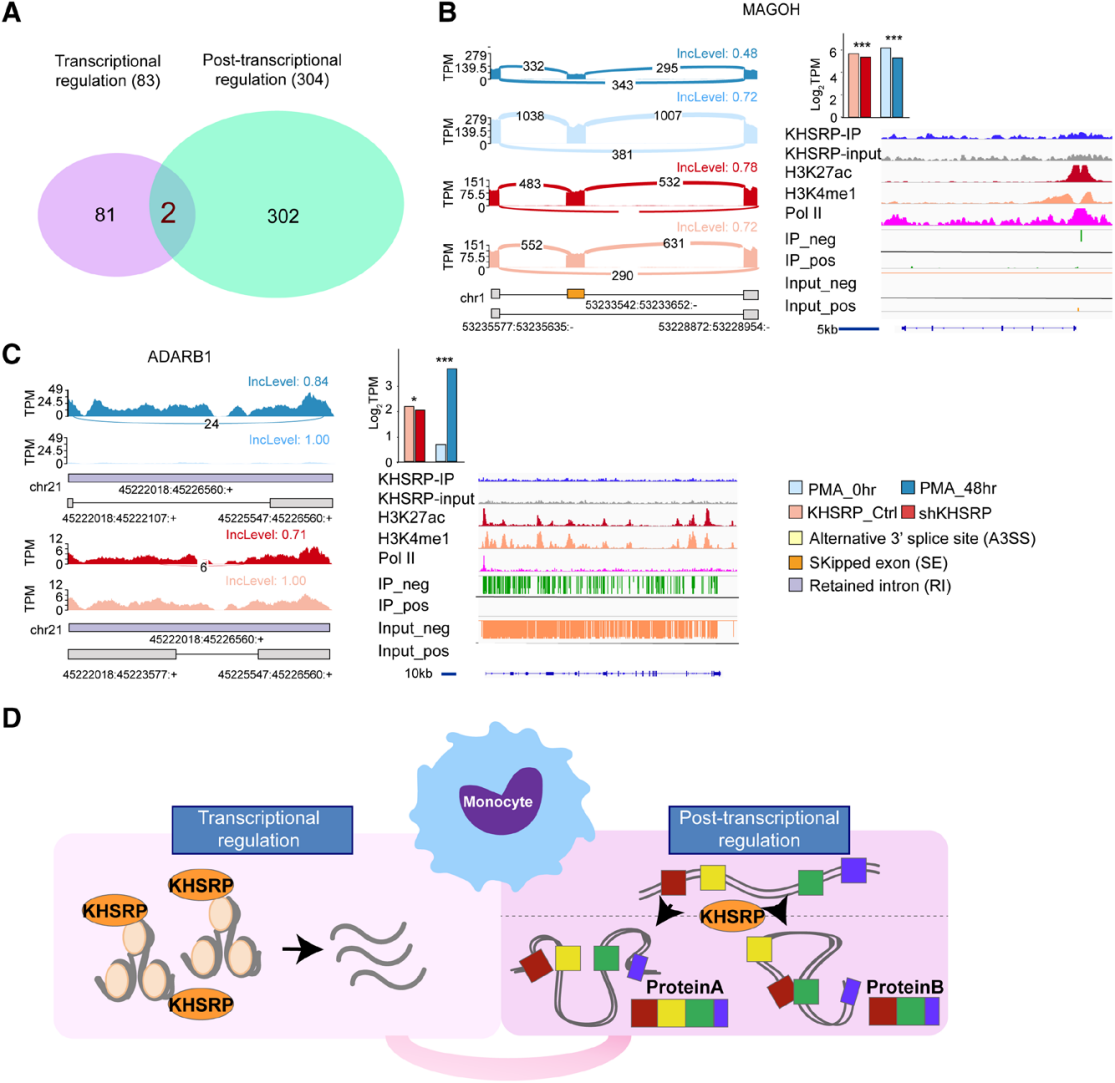
Characterization of potential co-transcriptional genes in monocytic differentiation. (A) Venn diagram showing the intersection of transcriptional regulatory genes and posttranscriptional regulatory genes in monocytic differentiation. (B, C) Sashimi plots showing differential AS events in both shKHSRP- and PMA-treated samples (left panel), and bar plot showing the expression levels of DEGs and genomic visualization of KHSRP, H3K27ac, H3K4me1, and Pol II ChIP-seq datasets and eCLIP signal on the indicated gene loci (right panel). (D) Schematic diagram of KHSRP’s transcriptional and posttranscriptional regulatory function during monocytic differentiation.

Based on our analysis, we identified 2 sets of KHSRP-regulated genes: 304 posttranscriptional target genes and 83 transcriptional target genes. Within the overlapping gene set, we obtained 4 candidate genes (adenosine deaminase RNA -specific B1 [*ADARB1*] and Mago homolog, exon junction complex subunit [*MAGOH*]) (Fig. 6A), which may be collaboratively regulated by KHSRP at both the transcriptional and posttranscriptional levels during monocyte differentiation (Fig. 6B–C). Sashimi plots showing the DASEs in both the KHSRP knockdown and PMA-treated samples (left panel) combined with the CLIP enrichment and KHSRP, H3K4me3, and Pol II colocalized signals are provided in Fig. 6B and C. MAGOH regulates the transcriptional activity of STAT3 by interfering in the formation of the STAT3/Y14 complex. *MAGOH* RNA expression was reduced following KHSRP knockdown, indicating that the expression of this gene is repressed during monocytic differentiation and is transcriptionally activated by KHSRP. In addition, the inclusion of exon X from *MAGOH* was decreased during monocytic differentiation and increased following KHSRP knockdown (Fig. 6B). The RNA-specific adenosine deaminase ADAR2 edits RNA by adenosine-to-inosine (A-to-I) deamination (Fig. 3C).^43,44^ RNA editing had been reported to be an important co-transcriptional RNA modification in mammals, and defects in this process are associated with human diseases.^45^ Moreover, ADAR2 not only plays an important role in the recoding of specific transcripts but also influences DNA repair that is dependent on ADAR2-editing of DNA:RNA hybrids to ease their dissolution.^46^ *ADARB1* was more highly expressed after PMA treatment but was repressed in shKHSRP-treated THP-1 cells. In addition, we identified differences in the alternative intron of *ADARB1* after PMA and shKHSRP treatment, in that the 5’ splice site differed while the 3’ splice site was the same.

Taken together, our results showed that KHSRP regulates monocytic differentiation through the expression control of several hematopoietic genes at both the transcriptional and posttranscriptional levels. More importantly, combining their DNA- and RNA-binding capacities, KHSRPs are likely to function collaboratively to regulate the transcription and RNA splicing of *MAGOH* and *ADARB1* in monocytic cells.

## 3. DISCUSSION

The subcellular localization of proteins can indicate their key functionalities. Therefore, the identification of the subcellular position of proteins is important to achieve a deeper understanding of their function. It is clear that RBPs function at the posttranscriptional level to regulate different stages of the gene expression process, including RNA splicing, RNA modification, RNA transport, RNA editing, RNA decay, and mRNA translation. The world of RBP research has expanded rapidly as a result of the proteome-wide identification of proteins involved in RNA binding and function, and their association with chromatin and DNA has been reported. For example, Lin28A binds DNA in the proximity of transcription start sites and recruits Tet1 to regulate gene transcription; WDR43 is recruited to gene promoters by nascent RNAs, leading to the release of Pol II and facilitating transcriptional elongation; while HnRNPU helps maintain the 3D structure of chromatin through oligomerization with chromatin-associated RNAs. In our previous study, we revealed the accumulation of such RBPs in the nuclear chromosomes (Che-RBPs) and characterized the transcriptional activation role of QKI5 during monocytic differentiation. Therefore, it seems that the chromatin-binding capacity of RBPs may represent a previously under-appreciated layer of gene expression regulation. What remains unclear is whether these Che-RBPs have both transcriptional and posttranscriptional regulatory roles, and if so, how they coordinate their functions at these 2 levels. Here, we revealed that KHSRP co-regulates the expression of several genes, both through transcriptional activation and post-transcriptional splicing, extending our understanding of RBP functions and suggesting that the dual RNA-/DNA-binding capacity has been underestimated and may, in fact, be a common attribute of such RBPs.

KHSRP is a multifunctional RBP that has been implicated in mRNA decay, AS, and miRNA biogenesis. Studies have demonstrated that the ability of KHSRP to posttranscriptionally regulate the expression of a variety of genes is required for cell-fate decisions, tissue regeneration, immune responses, lipid metabolism, and DNA damage responses. Our previous study focusing on RBPs that are functional during human monopoiesis revealed the essential effects of KHSRP on myeloid differentiation through the facilitation of miR-129 maturation. KHSRP has also been reported to regulate gene-specific splicing events in hematopoietic erythroid cells and demonstrated to regulate immune responses. Soonthornvacharin et al suggested that KHSRP is a regulator of the innate immune response to pathogenic challenges. As a negative regulator of antiviral signaling, KHSRP associates with the regulatory domain of RIG-I, reduces vital RNA associations with RIG-I during viral infection, and represses RIG-I activation.^47^ Moreover, KHSRP has been described as a direct negative regulator of type I IFN mRNA stability,^48^ and deletion of its expression leads to T-cell defects.^25^ By combining KHSRP eCLIP-seq and RNA-seq data from monocytes in this study, we showed that KHSRP modulates the alterative splicing of several essential monocytic genes, such as *PTK2B*, *PUM1*, and *LMO2*. We also revealed that KHSRP occupies specific genomic sites and activates the transcription of genes such as *CDC25A*, *HMGB1*, *KIT*, *NEAT1*, *PBX1*, and *PLD1*. Additionally, our results indicated that KHSRP functions collaboratively to regulate the transcription and RNA splicing of genes such as *MAGOH* and *ADARB1* in monocytic cells.

KHSRP is categorized as a DNA- and RBP (DRBP) that confers the capacity for powerful, coordinated control of gene expression and the ability to generate both immediate effects (by regulating RNA turnover) and long-lasting effects (by regulating transcription). One of the earliest known DRBPs, the glucocorticoid receptor, is a steroid hormone receptor shown to control the transcription of inflammatory genes and destabilize the mRNA of pro-inflammatory genes through direct RNA binding. A second example, NF90, is a versatile DRBP playing important roles in T-cell activation through the direct binding of DNA, mRNA, and miRNA, through which it controls transcription, regulates mRNA turnover and translation, and affects miRNA processing, respectively. These examples illustrate that DRBPs regulate gene expression at the transcriptional and post-transcriptional levels through their diverse DNA- and RNA-binding capacities.

## 4. MATERIALS AND METHODS

### 4.1. Availability of data and materials

ChIP-seq datasets of histone modifications and Pol II were downloaded from NCBI BioProject ID: PRJNA510375 (H3K27ac ChIP-seq datasets),^49^ PRJNA295216 (H3K4me3 ChIP-seq datasets),^50^ PRJNA63443 (H3K27me3, H3K79me2, and H3K36me3 ChIP-seq datasets),^51^ and PRJNA382164 (Pol II ChIP-seq datasets).^52^ The ChIP-seq, CLIP-seq, and RNA-seq datasets for KHSRP were downloaded from the Gene Expression Omnibus, accession number GSE161943 (Supplementary Table 1, https://doi.org/10.5281/zenodo.6496559).^7^

### 4.2. Knockdown/RNA-seq data processing

RNA-seq reads were aligned to the *Homo sapiens* genome (Ensembl GRCh38.p5) using TopHat2^53^ in the PE mode with default parameters, and uniquely mapped reads were retained for further analysis and filtered by SAMtools. HTSeq counts^54^ were used to calculate gene counts, and transcripts per million normalization was performed using in-house scripts. DEG analysis was conducted using DESeq2.^55^ DEGs were identified based on a *P* value of <.05 and |log2(fold change)| > log2 (1), and all genes with non-zero counts in any sample were considered.

Differential AS events were analyzed using rMATS (v 4.0)^56^ based on the knockdown replicate bam files and their control replicate bam files within the *H. sapiens* genome (Ensembl GRCh38.p5) annotation file. Five types of differential AS events (DASEs) were reported: SEs, MXEs, A3SSs, A5SSs, and RIs. DASEs with abs (IncLevelDifference) of >.05, a *P* value of <.05, and a false-discovery rate (FDR) of <.1 were identified as significant.

### 4.3. ChIP-seq data processing

ChIP-seq datasets of histone modifications (H3K4me3, H3K27ac, H3K27me3, H3K79me2, H3K36me3) and Pol II were downloaded from the European Bioinformatics Institute (http://wwwdev.ebi.ac.uk/).^57^ Details of these data sources are listed in the “Availability of data and materials” section.

Overall ChIP-seq dataset reads were aligned to the *H. sapiens* genome (Ensembl GRCh38.p5) using Bowtie2 in the PE mode with default parameters.^58^ SAMtools^59^ was used for further analysis, and reads with mapping quality scores >30 were retained. Two biological replicates were merged to create the “Tag Directory” file by “makeTagDirectory.” Peak finding and downstream data analyses were performed using “findPeaks” by HOMER.^60^ KHSRP ChIP-seq datasets were analyzed using the “factor” mode with the parameter “-tbp 1 -inputtbp 1 -F 2.5 -P .00001 -L 2.5 -LP .02 -ntagThreshold 3.5”. To identify histone modification in ChIP-seq and Pol II ChIP-seq datasets, we used the “histone” and “factor” modes, respectively, with default parameters.

The all-in-one program “annotatePeaks.pl” of HOMER was used to predict KHSRP DNA-binding sites in the genomic region. The repeatability of 2 biological replicates was evaluated according to the Pearson correlation coefficient, with read coverages for genomic regions per 1000 bp, which was generated by the “getPeakTags” program of HOMER. Histone modifications and the Pol II ChIP fragment depth around KHSRP promoter peaks (from −3000 bp to +3000 bp) were predicted by “annotatePeaks.pl” in HOMER. Overlapping peaks between each group of ChIP-seq datasets were identified using “intersectBed” in BEDTools.^61^ Integrative Genomics Viewer software^62^ was used to visualize the predicted gene loci in the KHSRP ChIP-seq datasets. MEME-ChIP tools in the MEME online suite (http://meme-suite.org/) were used for KHSRP DNA-binding motif discovery, coupled with an e-value to determine motif enrichment and significance.^63^ Transcription factor (TF) motifs in the vicinity of the KHSRP motif were determined using SpaMo tools in the MEME online suite, coupled with an e-value to determine motif enrichment and significance.^8^

### 4.4. eCLIP-seq data processing

The KHSRP eCLIP-seq datasets were processed in accordance with previous studies, and the eCLIP-seq data processing pipeline is available at https://github.com/YeoLab/eclip. Raw reads with distinct inline barcodes were demultiplexed using in-house scripts, and the 10-mer random sequence was appended to the read name in the bam file for later use. Low-quality reads and adapter sequences were trimmed by cutadapt. Repetitive reads were removed by aligning reads with human repetitive element sequences in the RepBase database (https://www.girinst.org/) by STAR. Cleaned reads were mapped to the *H. sapiens* genome (Ensembl GRCh38.p5) by STAR.^64^ Duplicate PCR reads were removed by in-house scripts based on their sharing of identical random sequences. Two biological replicates were merged using “merge” in SAMtools for subsequent analysis. Peak calling and downstream data analyses were performed using Clipper software.^65^ Peak normalization was performed using “Peak_input_normalization_wrapper.pl” tools, available at “https://github.com/YeoLab/eclip”. CLIP-seq peaks were filtered based on a *P* value of < 10e-3 and fold change of > 4.

Enrichment of KHSRP RNA-binding sites in the human genomic region was calculated by ChIPseeker in the R package.^66^ The repeatability of 2 biological replicates was evaluated by Pearson correlation coefficient, with read coverages for genomic regions per 1000 bp, which were generated by “multiBamSummary” in Deeptools.^67^ The relative distance between ChIP-seq peaks and neighboring CLIP-seq peaks was determined by “closest” in BEDTools. The MEME-ChIP tool in the MEME online suite (http://meme-suite.org/) was used for KHSRP RNA binding motif discovery, coupled with an e-value to determine motif enrichment and significance with the standard RNA alphabet.^63^

### 4.5. Gene set enrichment analysis

Gene ontology functional enrichment analysis was performed by Metascape (http://metascape.org/),^68^ which applies the standard accumulative hypergeometric statistical test to identify ontology terms.

## ACKNOWLEDGMENTS

This work was supported by the National Key Research and Development Program of China (2019YFA0801800, 2021YFA1102400, 2019YFA0802600, and 2021YFA0805703); the National Natural Science Foundation of China (81530007, 31900072, 31725013, 82022001, 82122005, 81970103, and 81970101); and CAMS Innovation Fund for Medical Sciences (2021-I2M-1-019 and 2021-I2M-1-040).
